# Fear, Resistance, or Anticipation? Older Truck Drivers’ Reactions to the Adoption of Automated Vehicles

**DOI:** 10.1093/geroni/igab046.3491

**Published:** 2021-12-17

**Authors:** Amy Schuster, Jenna Van Fossen, Danielle Sperry, Shelia Cotten

**Affiliations:** 1 Clemson University, Clemson, South Carolina, United States; 2 Michigan State University, East Lansing, Michigan, United States

## Abstract

The forecasted adoption of autonomous vehicles (AVs) will lead to major changes to the job of truck driving. These changes may be particularly challenging for drivers, as the population of truck drivers skews much older than that of other occupations. In this study we sought to understand truck drivers’ attitudes towards AVs and the longevity of their job. We conducted focus groups with truck drivers, their supervisors, and upper-level managers of trucking companies. We relate supervisors’ and managers’ experiences working with drivers through the rollout of new technologies to further understand drivers’ initial reactions to automation and how their attitudes may develop. Based on qualitative open coding our analysis uncovered two overarching themes. The first theme is the unknown. With AVs, companies expect that experience will be less important, so they can hire younger workers. In response, drivers have expressed fear of being displaced and anxiety over the uncertainty of not knowing how their jobs will be affected. The second theme is adaptability, and desire to adapt. Older drivers have expressed resistance to adapting to AVs and to their job changing. Concerningly however, managers envision the need for a driving workforce that has experience working with technology and is adaptable. Our study identifies key challenges concerning older workers’ reactions and career decisions in response to automation. Accounting for driver reactions to AVs is necessary not only to build theory and understanding on worker reactions to automation, but also for workforce planning and to support employees, particularly older workers.

